# Effects of naloxone and diazepam on blood glucose levels in tramadol overdose using generalized estimating equation (GEE) model; (an experimental study)

**DOI:** 10.1186/s12902-021-00847-x

**Published:** 2021-09-06

**Authors:** Samaneh Nakhaee, Khadijeh Farrokhfall, Ebrahim Miri-Moghaddam, Masoumeh Askari, Alireza Amirabadizadeh, Mohsen Foadoddini, Omid Mehrpour

**Affiliations:** 1 Medical Toxicology and Drug Abuse Research Center (MTDRC), Birjand University of Medical Sciences (BUMS), Birjand, Iran; 2 Cardiovascular Diseases Research Center, Birjand University of Medical Sciences, Birjand, Iran; 3 Mel and Enid Zuckerman College of Public Health, University of Arizona, Tucson, AZ USA

**Keywords:** Tramadol, Diazepam, Naloxone, Blood glucose, Overdose

## Abstract

**Background:**

Tramadol is a synthetic opioid and poisoning is increasing around the world day by day. Various treatments are applied for tramadol poisoning. Due to the unknown effects of tramadol poisoning and some of its treatments on blood glucose levels, this study was conducted to investigate the overdose of tramadol and its common treatments (naloxone, diazepam), and their combination on blood glucose levels in male rats.

**Methods:**

This study was conducted in 45 male Wistar rats. The animals were randomly divided into five groups of 9. They received a 75 mg/kg dose of tramadol alone with naloxone, diazepam, and a combination of both of these two drugs. On the last day, animals’ tail vein blood glucose levels (BGL) were measured using a glucometer at different times, including before the tramadol injection (baseline) and 1 hour, 3 hours, and 6 hours after wards. The rats were anesthetized and sacrificed 24 h after the last injection. Blood samples were then taken, and the serum obtained was used to verify the fasting glucose concentration. Data were analyzed using SPSS software at a significance level of 0.05 using a one-way analysis of variance (ANOVA) and a generalized estimating equation (GEE).

**Results:**

According to the GEE model results, the diazepam-tramadol and naloxone-diazepam-tramadol groups showed blood glucose levels five units higher than the tramadol group (*p* < 0.05). The diazepam-tramadol group had significantly higher blood glucose levels than the naloxone-tramadol group (*p* < 0.05). The mean blood glucose levels before the intervention, 3 hours and 6 hours after the injection of tramadol did not differ between the groups, but the blood glucose levels 1 hour after the injection of tramadol in the group of naloxone-tramadol were significantly lower than in the control group (*p* < 0.05). Blood glucose levels did not differ between the groups 24 h after injection of tramadol.

**Conclusion:**

The results of the present study showed tramadol overdose does not affect blood glucose levels. The diazepam-tramadol combination and the diazepam-naloxone-tramadol combination caused an increase in blood glucose levels.

## Background

Tramadol is widely used throughout the world. This has led to an increase in the number of cases of poisoning, side effects and deaths [1]. Several cases of toxicity and abuse caused by this drug have been reported in the medical literature [2, 3]. Tramadol is an effective pharmaceutical drug for moderate to severe pain relief that affects the transmission of pain impulses through the μ-opioid receptor and inhibits norepinephrine and serotonin reuptake [4]. The most important side effects of tramadol poisoning include cognitive impairment, agitation, respiratory depression, seizures, respiratory distress, serotonin syndrome, kidney failure, and rhabdomyolysis [5]. Tramadol overdose generally leads to self-limiting, generalized, tonic-clonic seizure, most frequently occurring during 4–6 h after ingestion [6]. Seizure is more common in tramadol poisoning than with other opioids. Although the exact underlying mechanism of tramadol-induced seizure activity is not clarified yet, it has been suggested the seizurogenic effect of tramadol is possibly mediated via decreased serotonin reuptake and inhibitory effects on γ-aminobutyric acid (GABA) pathways [7]. Tramadol overdose-induced blood glucose changes is another potential side effect that has received insufficient attention. These changes may be among the predisposing factors for seizures in patients presenting with tramadol overdose [8]. Altered blood glucose has been associated with changes in hepatic gluconeogenesis and peripheral glucose uptake [9]. Serial blood glucose monitoring has been instructed to detect and manage hypoglycemia and hyperglycemia in tramadol overdose [8]. A recent systematic review disclosed that hypoglycemia is more likely to occur than hyperglycemia after tramadol administration with therapeutic use and overdose. Also, all studies on tramadol use in diabetes documented hypoglycemia [1].

In tramadol poisoning cases several treatments are applied, however, there are some debates and challenges regarding the exact indications for using these treatments. The effects of these treatments have been investigated in tramadol-induced seizures [10–13], but their effects on blood glucose levels are less well studied. Naloxone can be used as an opioid antagonist to prevent respiratory depression in tramadol overdose cases [10] Benzodiazepines are other drugs used in seizures caused by tramadol poisoning [6]. Several studies on these two drugs’ effects on glucose metabolism have reported conflicting results [14–19]. To our knowledge, no studies have examined their effects on BGL when used to treat tramadol poisoning. This study was conducted to investigate tramadol, naloxone, diazepam, and their combination in male rats’ blood glucose.

## Methods

This study was conducted in 45 male Wistar rats (body weight 200–250 g, 12 weeks) under standard laboratory conditions (constant room temperature (22 ± 2 °C), light / dark periods of 12 h, and free access to food and water). The rats were purchased from the animal experimental center of Birjand University of Medical Sciences (Birjand, Iran).

The Institutional Animal Ethics Committee approved all animal experiments by Birjand University of medical sciences (Birjand, Iran) (code: IR.BUMS.REC.1397.194). This study was conducted in accordance with international and institutional guidelines for the care and use of animals. The ARRIVE (Animal Research: Reporting of in Vivo Experiments) guidelines for the standard care and animals’ use were followed.

Naloxone, diazepam, and pentobarbital were obtained from Sigma-Aldrich and tramadol from Temad (Iran, Karaj). Diazepam and naloxone were dissolved in DMSO and tramadol in normal saline. The DMSO concentration for this study was 2%. The injection volume was 1 ml per kg of body weight. DMSO was prescribed in an inert range for behavioral [20] and experimental [21] animal studies.

Healthy animals with typical behavior and activity from the same species, genders, weighing 200 to 250 g, and ages of 12 weeks were included. Rats used previously in other experiments were not included. Exclusion criteria were considered death during experiments and aberrant behavior. After 1 week of adaptation to laboratory conditions, the animals were randomly divided into five groups, to include nine animals in each group: the control group, the tramadol group, the naloxone-tramadol group, the diazepam-tramadol group, and finally the naloxone-diazepam-tramadol group. The calculation of sample size is based on the “resource equation approach” design for animal studies using the formula of N = (10/*k* + 1) × *k*, where *N* = total number of subjects, *k* = number of groups [22].

- The control group (the first group) received intraperitoneally (IP) over 14 days, an injection of 0.9% normal saline.
- The tramadol group (the second group) received an intraperitoneal injection of 25 mg/kg of tramadol for 13 days. Subsequently, on day 14, they received an intraperitoneal injection, 75 mg/kg of tramadol.
- The naloxone group (the third group) also received an intraperitoneal injection of 25 mg/kg of tramadol for 13 days. Subsequently, on day 14, they were administered 75 mg/kg of tramadol by acute intraperitoneal injection, which was accompanied by naloxone (2 mg/kg) intravenously 15 min after the injection of tramadol. After this, they received naloxone (4 mg/kg) for up to 6 h as an hourly injection [23].
- The diazepam group (the fourth group) for 13 days received 25 mg/kg of tramadol intraperitoneally. Subsequently, on day 14, they were administered tramadol, also intraperitoneally, 75 mg/kg of acute dose. Then, 15 min after the tramadol injection, they were administered 1.77 mg/kg of diazepam intraperitoneally.
- The naloxone-diazepam group (fifth group) received 25 mg/kg of tramadol intraperitoneally over a period of 13 days. On day 14, they were injected intraperitoneally with tramadol at an acute dose of 75 mg/kg, an injection of 1.77 mg/kg of intraperitoneal diazepam, and intravenous naloxone at a dose of 2 mg/kg. Finally, for up to 6 h, they received an injection of 4 mg/kg of naloxone every hour.

On the last day, the rat tail vein blood glucose levels were measured using a glucometer by making a 2 mm transverse incision at the end of the animal’s tail. On the fourteenth day, blood glucose was measured in all groups at different times, including before the injection of tramadol (baseline) and 1 hour, 3 hours, and 6 hours after tramadol injection. The rats were also clinically monitored for 6 h on the final experimental day, and the number of seizures in different groups were recorded using a video recorder. The toxicity of tramadol and its dose selection were based on the onset of typical tramadol overdose features in the rats; our model exhibited tramadol-induced seizures and decreased level of consciousness similar to that reported in humans [24–26]. The animal model for tramadol overdose was based on how patients who use tramadol chronically may intentionally or accidentally overdose. Rats were anesthetized and sacrificed by intraperitoneal administration of 60 mg/kg of pentobarbital 24 h after the last injection (fasting for 12 to 14 h). Blood samples were then taken. Serum was obtained by centrifugation of the samples and stored until the parameters were measured. The serum achieved was used to evaluate the fasting glucose concentration and creatinine levels. The experimenter was aware of the rat group, but a researcher blinded to the group allocation carried out statistical analysis and outcome assessment.

After collection, the data were entered into the SPSS software (version. 16). Using the Shapiro Wilk test, the hypothesis of normality of the quantitative variables was examined. One-way analysis of variance (ANOVA) and Bonferroni’s post hoc test was used to compare means in various groups. The generalized estimation equation (GEE) model was used to analyze the longitudinal variables of blood glucose at different times. The longitudinal variable was entered as a dependent variable in the GEE model and the group as an explanatory variable. The level of significance was considered less than 0.05.

## Results

**Fig. 1:**
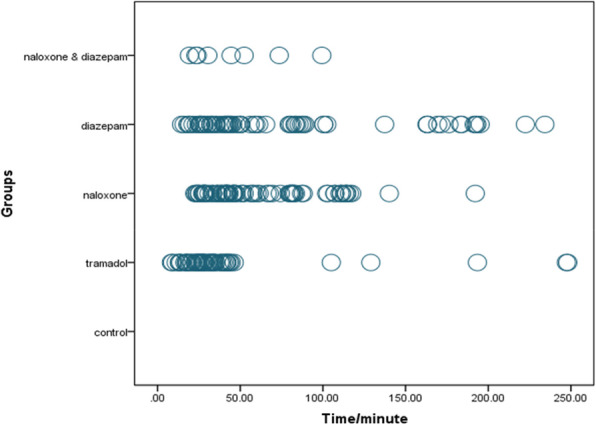
Time-course of seizure occurrence in the different treatment groups in Wistar rats of intraperitoneal (IP) 0.9%saline for14 days (control group), IP 25 mg/kg tramadol for 13 days and 75 mg/kg on the 14th day (tramadol group), IP 25 mg/kg tramadol for 13 days and 75 mg/kg tramadol + naloxone intravenous (IV) 2 mg/kg bolus followed by injection of 4 mg/kg/h 14th day (naloxone group), IP 25 mg/kg tramadol for13 days and 75 mg/kg tramadol + 1.77 mg/kg diazepam 15 min after tramadol injection on the 14th day (diazepam group), IP 25 mg/kg tramadol for 13 days and 75 mg/kg tramadol + naloxone intravenous (IV) 2 mg/kg bolus followed by injection of 4 mg/kg/h + 1.77 mg/kg diazepam 15 min after tramadol injection on the 14th day

**Fig. 2:**
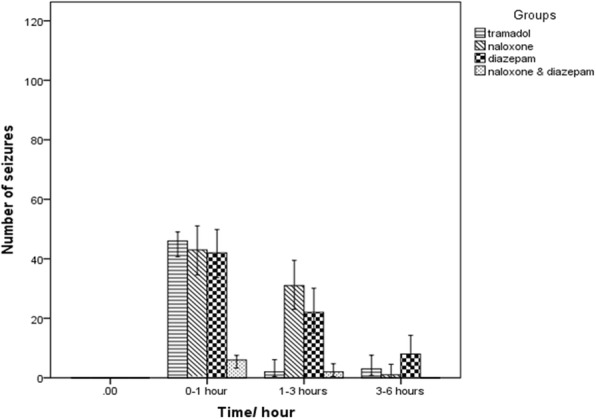
The number of seizures occurring during 6 h in the different groups in Wistar rats of intraperitoneal (IP) 0.9%saline for14 days (control group), IP 25 mg/kg tramadol for 13 days and 75 mg/kg on the 14th day (tramadol group), IP 25 mg/kg tramadol for 13 days and 75 mg/kg tramadol + naloxone intravenous (IV) 2 mg/kg bolus followed by injection of 4 mg/kg/h 14th day (naloxone group), IP 25 mg/kg tramadol for13 days and 75 mg/kg tramadol + 1.77 mg/kg diazepam 15 min after tramadol injection on the 14th day (diazepam group), IP 25 mg/kg tramadol for 13 days and 75 mg/kg tramadol + naloxone intravenous (IV) 2 mg/kg bolus followed by injection of 4 mg/kg/h + 1.77 mg/kg diazepam 15 min after tramadol injection on the 14th day

One death occurred in the naloxone-diazepam-tramadol group during the study. Before and after the study, the rats’ mean weight did not differ between the groups (*p* > 0.05). The number of seizures for each animal was counted, and then the average of all seizures occurring in each group was calculated and compared (χ2 = 13.2, *p* = 0.01). The number of seizures in all experimental groups was significantly higher than the control group. The naloxone-diazepam significantly decreased the incidence of seizures compared to the tramadol group (*P* < 0.05) (Figs. 1 and 2).

**Fig. 3:**
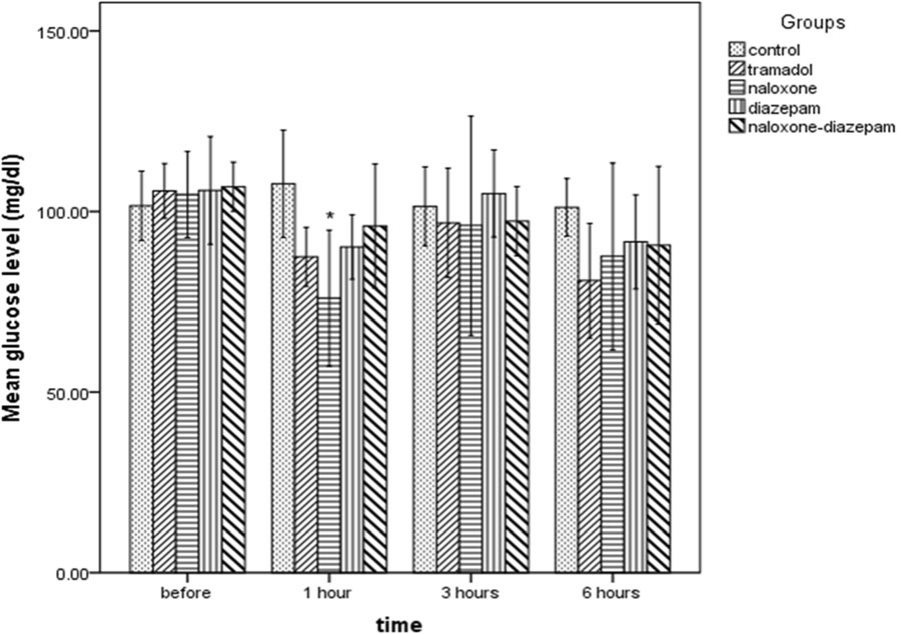
Comparison of blood glucose levels in different times in Wistar rats of intraperitoneal (IP) 0.9%saline for14 days (control group), IP 25 mg/kg tramadol for 13 days and 75 mg/kg on the 14th day (tramadol group), IP 25 mg/kg tramadol for 13 days and 75 mg/kg tramadol + naloxone intravenous (IV) 2 mg/kg bolus followed by injection of 4 mg/kg/h 14th day (naloxone group), IP 25 mg/kg tramadol for13 days and 75 mg/kg tramadol + 1.77 mg/kg diazepam 15 min after tramadol injection on the 14th day (diazepam group), IP 25 mg/kg tramadol for 13 days and 75 mg/kg tramadol + naloxone intravenous (IV) 2 mg/kg bolus followed by injection of 4 mg/kg/h + 1.77 mg/kg diazepam 15 min after tramadol injection on the 14th day. Data are the mean ± SD. *p < 0.05 compared to control group

This study showed that a low dose of tramadol administration for thirteen days did not affect glucose levels. The ANOVA test results showed the mean blood glucose levels before the intervention (F = 0.25, *p* = 0.9), 3 hours (F = 0.31, *p* = 0.8), and 6 hours (F = 1.17, *p* = 0.3) after the injection of tramadol did not differ between the groups. Still, 1 hour after the tramadol injection, there was a significant difference between groups (F = 4.65, *p* = 0.005), so that the glucose level in the naloxone-tramadol group was significantly lower than in the control group based on the Bonferroni’s post hoc test (*p* = 0.002). There was no significant difference between the other groups (*p* > 0.05) (Fig. 3).

**Fig. 4:**
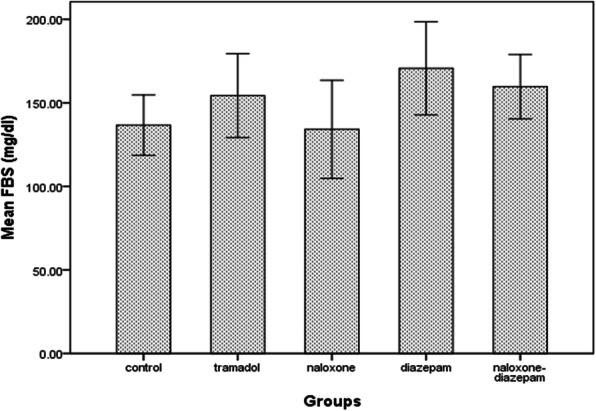
Comparison of fasting blood glucose levels in different groups after 24 h in Wistar rats of intraperitoneal (IP) 0.9%saline for14 days (control group), IP 25 mg/kg tramadol for 13 days and 75 mg/kg on the 14th day (tramadol group), IP 25 mg/kg tramadol for 13 days and 75 mg/kg tramadol + naloxone intravenous (IV) 2 mg/kg bolus followed by injection of 4 mg/kg/h 14th day (naloxone group), IP 25 mg/kg tramadol for13 days and 75 mg/kg tramadol + 1.77 mg/kg diazepam 15 min after tramadol injection on the 14th day (diazepam group), IP 25 mg/kg tramadol for 13 days and 75 mg/kg tramadol + naloxone intravenous (IV) 2 mg/kg bolus followed by injection of 4 mg/kg/h + 1.77 mg/kg diazepam 15 min after tramadol injection on the 14th day. Data are the mean ± SD

According to this study, the mean blood glucose levels 24 h after tramadol injection did not differ between the groups (F = 2.43, *p* = 0.07) (Fig. 4).

The diazepam-tramadol and naloxone-diazepam-tramadol groups showed blood glucose levels five units higher than the tramadol group based on the GEE model results (*p* = 0.01). Furthermore, the diazepam-tramadol group had significantly higher blood glucose levels than the naloxone-tramadol group (B = 7.04, *p* = 0.004). The other groups did not show significant effects on blood glucose compared to each other (*p* > 0.05) (Table 1).

**Table 1:** Comparison of glucose levels in the different groups based on the GEE model

	B	SE	Wald	*p*-value
Tramadol vs Control	−10.25	5.24	3.81	0.05
Naloxone-tramadol vs Control	−11.86	6.45	3.38	0.07
Diazepam-tramadol vs Control	−4.82	4.60	1.09	0.29
Naloxone-Diazepam-tramadol vs Control	−5.15	3.37	2.33	0.13
Naloxone-tramadol vs Tramadol	−1.61	3.23	0.25	0.62
Diazepam-tramadol vs Tramadol	5.43	2.10	6.68	0.01
Naloxone-Diazepam-tramadol vs Tramadol	5.09	2.09	5.91	0.01
Diazepam-tramadol vs Naloxone-tramadol	7.04	2.47	8.10	0.004
Naloxone-Diazepam-tramadol vs Naloxone-tramadol	6.71	3.89	2.97	0.08
Naloxone-Diazepam-tramadol vs Diazepam-tramadol	−0.36	2.40	0.02	0.88

**Fig. 5:**
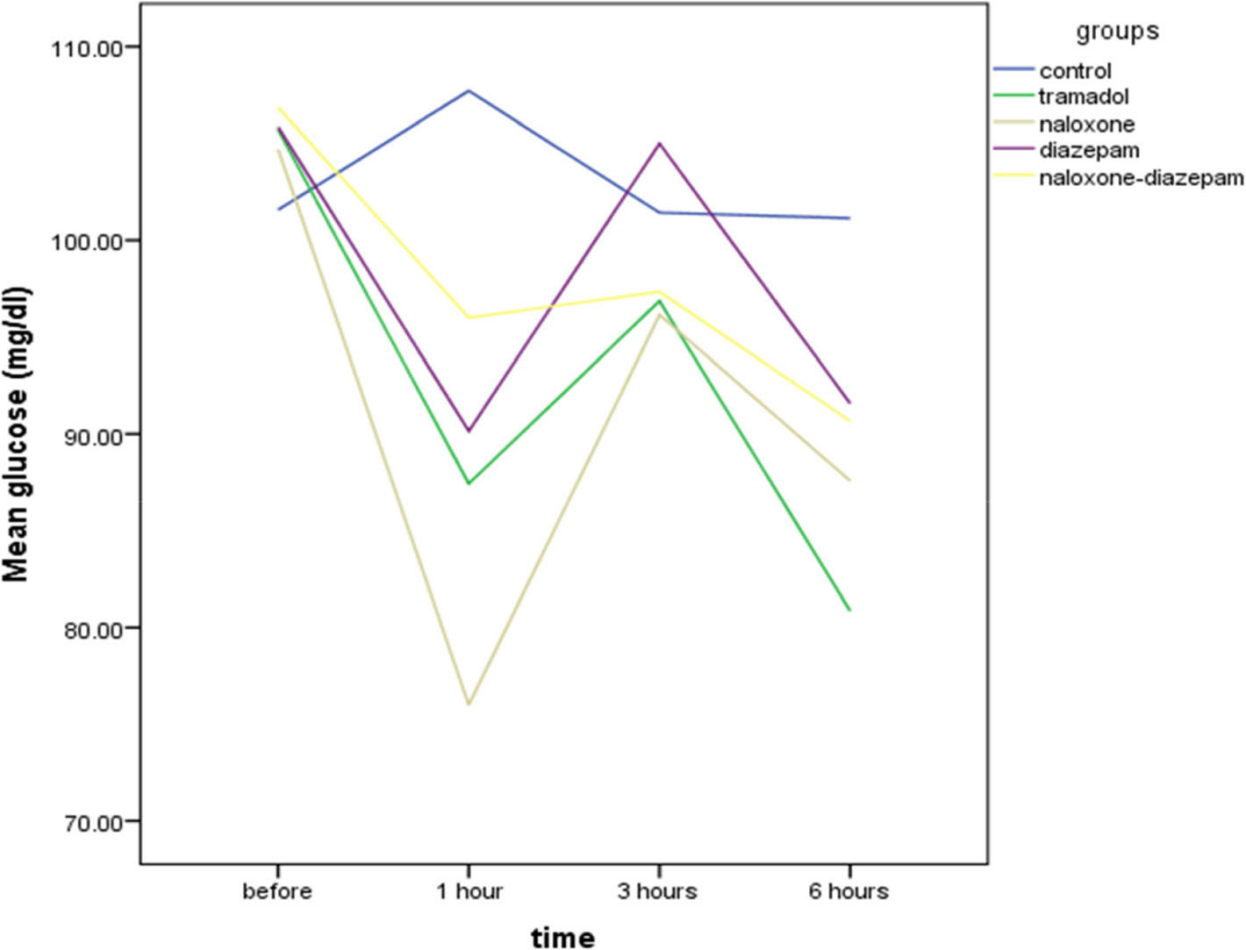
Time-course of average glucose levels in the different treatment groups in Wistar rats of intraperitoneal (IP) 0.9%saline for14 days (control group), IP 25 mg/kg tramadol for 13 days and 75 mg/kg on the 14th day (tramadol group), IP 25 mg/kg tramadol for 13 days and 75 mg/kg tramadol + naloxone intravenous (IV) 2 mg/kg bolus followed by injection of 4 mg/kg/h 14th day (naloxone group), IP 25 mg/kg tramadol for13 days and 75 mg/kg tramadol + 1.77 mg/kg diazepam 15 min after tramadol injection on the 14th day (diazepam group), IP 25 mg/kg tramadol for 13 days and 75 mg/kg tramadol + naloxone intravenous (IV) 2 mg/kg bolus followed by injection of 4 mg/kg/h + 1.77 mg/kg diazepam 15 min after tramadol injection on the 14th day

All study groups, except the control group showed a similar pattern of blood glucose changes at different times such that 1 hour after tramadol injection they all showed a decrease in blood glucose levels and these values ​​returned nearly close to baseline levels 3 hours after injection (Fig. 5). One-way analysis of variance showed that mean creatinine levels were significantly different between groups (F = 4.5, *p* = 0.007). Bonferroni’s post hoc test showed Tramadol had no significant effects on creatinine levels. Still, the naloxone-diazepam-tramadol significantly increased the mean creatinine levels compared to control (0.52, ±0.1 vs. 0.35 ± 0.04, *p* = 0.01) and tramadol (0.52, ±0.1 vs. 0.36 ± 0.05, *p* = 0.02) groups.

## Discussion

This study showed that tramadol overdose does not affect blood glucose levels although its changes are aimed at lowering blood glucose. Conflicting results have been reported in several studies. Some studies have reported hypoglycemia in tramadol poisoning [5, 8, 27–36] and others have reported elevated blood glucose levels in these individuals [31, 37, 38]. Consistent with our results, several studies have shown normal blood glucose levels in people with tramadol poisoning [4, 8, 31, 34, 36, 39, 40].

The different results in different studies can be attributed to differences in the samples studied, different doses of tramadol, different measurement times of BGL, and various methods of administration. The proposed mechanism for tramadol’s hypoglycemic effects is the activation of opioid receptors by tramadol, resulting in glucose utilization in peripheral tissues [41].

Previous reports indicate the opioid receptor is the main target involved in tramadol-induced hypoglycemia [42]. Some studies also showed serotonin could increase insulin levels, release beta-endorphins, and ultimately stimulate muscle glucose utilization [43, 44].

Catecholamines have been shown to directly suppress insulin secretion from the pancreas, causing glycogenolysis in the liver by stimulating the α-adrenergic receptor and ultimately causing hyperglycemia [45, 46]. Some studies showed a hyperglycemic effect of tramadol. For example, Kara et al. (2013) attributed the induction of tramadol-induced hyperglycemia to α2 adrenergic receptors’ activity, suggesting that monoamine pathways affect the analgesic properties of tramadol and may be involved in the development of drug-induced hyperglycemia [47]. Another result of this study was the increasing effect of diazepam-tramadol and naloxone-diazepam-tramadol on BGL. Diazepam is widely used as a sedative and as an anticonvulsant. According to some reports, the benzodiazepine receptor mediates its action by increasing synaptic GABA inhibition [48]. According to some reports, it prevents various stress-induced changes such as activation of the HPA axis [49]. Dexit et al. prescribed diazepam (0.6 mg/kg/day) to rabbits for 1 month and reported no effect on blood glucose levels [50].

In contrast, some studies have reported that diazepam increases blood glucose in humans and animals. For example, the results of a study in mice showed that administration of diazepam (13.3 mg/kg) resulted in an increase in blood glucose levels at different times after injection [15]. Another study in rabbits showed that diazepam (2 mg/kg/iv) could increase blood glucose levels [51].

Furthermore, another study in rats showed that diazepam could increase blood glucose levels in a dose-dependent manner [52]. Their research suggested that diazepam effects on peripheral glucose were the possible cause of benzodiazepine-related hyperglycemia [52]. In an experimental model, the 2α-adrenergic receptor antagonist could prevent diazepam-induced hyperglycemia. The results of this study suggested diazepam-induced hyperglycemia may be associated with adrenaline release from the adrenal gland [48].

The GABA-A receptor subunit expression has also been reported in the pancreas [53] and chromaffin cells of the adrenal medulla [54]. GABA can regulate the endocrine function of β cells through direct or indirect activation [55].

It is hard to explain the glycemic changes of diazepam at an animal stage of examination, and the results should be interpreted with caution at this stage.

The effects of diazepam also need to be interpreted in the context of tramadol poisoning due to drug interactions. The racial and dose-dependent characteristics of glucose homeostasis must be considered when interpreting the results of various studies. And we propose to investigate these treatments in several doses. Different single-dose therapies were used in tramadol overdose in this study. Additionally, future studies may consider providing a dose-response curve and other factors related to blood glucose homeostasis. Our study has limitations, including the fact that tramadol concentrations and its metabolites (M1, M2, and M5) were not measured. Also, in this study, vein blood glucose levels (BGL) were measured using a glucometer at different times. It may be insufficient for a complete and comprehensive elucidation of a scientific conclusion. It would be more beneficial if the effect of tramadol overdose on glucose infusion test (GIT) and more kidney function tests are considered in future studies.

The generalization of animals’ findings to humans should be performed cautiously due to this study’s empirical nature. Assume these treatments’ effects on blood glucose levels and glucose homeostasis in human tramadol overdose, the hyperglycemic effects of diazepam, and the hypoglycemic effects of naloxone in the early hours must be considered, especially for patients with diabetes.

## Conclusion

Our study results showed a low dose of tramadol administration; typically, thirteen days did not affect glucose levels. Tramadol overdose had minimal effects on blood glucose levels. Naloxone administration can also decrease blood glucose levels in the first hours after tramadol overdose, diazepam -tramadol combination and diazepam-naloxone- tramadol combination may increase blood glucose levels in the event of a tramadol overdose.

## Data Availability

The datasets are available from the corresponding author on formal and logic request.

## Abbreviations

GEE: Generalized estimating equation
GABA: γ-Aminobutyric acid
DMSO: Dimethyl sulfoxide
IP: Intraperitoneal
BGL: Blood glucose levels
ANOVA: One-way analysis of variance
